# The relationship between dietary patterns and rheumatoid arthritis: a case–control study

**DOI:** 10.1186/s12986-020-00502-7

**Published:** 2020-09-17

**Authors:** Shokufeh Nezamoleslami, Reza Ghiasvand, Awat Feizi, Mansour Salesi, Makan Pourmasoumi

**Affiliations:** 1 Department of Community Nutrition, School of Nutrition and Food Science, Isfahan University of Medical Sciences, Isfahan, Iran; 2 Department of Community Nutrition, School of Nutrition and Food Science, Isfahan University of Medical Sciences, P.O. Box 81745, Isfahan, Iran; 3 Department of Biostatistics and Epidemiology, School of Public Health, Isfahan University of Medical Sciences, Isfahan, Iran; 4 Department of Rheumatology, Isfahan University of Medical Sciences, Isfahan, Iran; 5 Gastrointestinal and Liver Diseases Research Center, Guilan University of Medical Sciences, Rasht, Iran

**Keywords:** Rheumatoid arthritis, Dietary patterns, Factor analysis, Incidence, Case–control

## Abstract

**Background and aim:**

A number of studies have investigated the effects of individual foods and/or nutrients on rheumatoid arthritis (RA), but research focusing on whole dietary patterns remains limited. The association of dietary patterns and rheumatoid arthritis is therefore not well elucidated. This study aims to determine existing relationships between major identified dietary patterns and RA.

**Methods:**

This matched case–control study was conducted on 297 individuals in Isfahan, Iran. The presence of RA was determined by an expert rheumatologist, based on the American College of Rheumatology definitions, 2010. A 168-item questionnaire was used to collect dietary data. Major dietary patterns were identified using the factor analysis method.

**Results:**

Two major dietary patterns, namely, healthy and western dietary patterns, were identified. Lower adherence to the healthy dietary pattern was associated with increased risk of RA (OR = 2.80; 95% CI 1.74–4.67; *P* < 0.001). The association remained significant even after taking other confounders into account (OR = 2.85; 95% CI 1.12–7.45; *P* = 0.03). A positively significant association was also observed between adherence to western dietary pattern and RA in the fully-adjusted final model (OR = 2.22; 95% CI 1.04–4.72; *P* = 0.03).

**Conclusions:**

The study suggests that there is an inverse association between adherence to a healthy dietary pattern and the odds of RA, and a positive significant relationship was found between western dietary pattern and RA. Further studies are required to confirm these findings.

## Introduction

Rheumatoid arthritis (RA) is a systemic autoimmune disease with effects on synovial membranes and joints as a result of inflammation. It leads to joints damage, muscle erosion, atrophy, and pain, and can also impair physical function [1–3]. The prevalence of RA among adults around the world is estimated as 0.5–1% [4]. It affects women 3–5 times more than men [5]. RA has been associated with increased healthcare costs, productivity losses, reduced quality of life, and lower life expectancy, resulting in substantial economic impacts on patients, their families, and society [6–9].

Both inherited and environmental variables are involved in the etiology of RA, although the main cause of RA has not been clearly elucidated. Several environmental factors may contribute to the etiology of RA, including unhealthy lifestyle choices, dietary risk factors, smoking, and infection [10].

Dietary intake is considered as a modifiable risk factor associated with RA, perhaps through mediating systemic inflammation. A number of studies have shown that higher consumption of protein and red meat, as well as lower intakes of vegetables, olive oil and vitamins, increased the risk of inflammatory polyarthritis or RA [11–14]. Several studies have reported that a Mediterranean diet might have beneficial effects in preventing or managing RA. In contrast, other studies did not replicate these results [15, 16]. The aforementioned studies mainly focused on single food items or nutrients, however. Considering food consumption in terms of dietary patterns can instead offer a more comprehensive view of the combined effects of nutrients and foods [17]. Due to the lack of studies assessing major dietary patterns in Middle Eastern countries, especially in Iranian populations, the current case–control study aimed to evaluate the relationship between dietary patterns and the likelihood of RA in Iranian adults.

## Methods

### Study population

In this matched case–control study, 297 participants (100 patients and 197 healthy controls) aged 19–69, were recruited in Isfahan, Iran. Each patient was paired with 2 healthy controls by gender. 100 patients newly diagnosed with RA were selected by an available-sampling methodology from Sepahan Clinic in Isfahan. RA diagnosis was made by a rheumatologist, based on the American College of Rheumatology 2010 classification criteria. RA onset was defined as the day of diagnosis, as stated in participant medical records. RA cases with a maximum of 1 year since diagnosis were included. In addition, 197 healthy subjects were randomly selected from the same clinic. Subjects were not included in the study if they had any history of chronic diseases, such as cardiovascular diseases, renal disease, liver diseases, or cancer, if they were on any specific diets or food prohibition schedules, or consumed alcohol. Participants with incomplete food frequency questionnaire (FFQ) were excluded, who answered less than 35 items of the FFQ. Those with unacceptable FFQ responses (energy intake < 800 kcal/day or > 4200 kcal/day) were also excluded [18]. All participants gave written informed consent. This study was approved by Research Council and the Ethics Committee of Isfahan University of Medical Sciences (397756) (IR.MUI.Research.REC.1398.120).

### Dietary assessment

Dietary information was obtained using a validated 168-item semi-quantitative food frequency questionnaire (FFQ) [19]. For each food item, participants were asked how often, on average, over the previous year, they had consumed the food, according to a commonly used unit or portion size. Frequency choices (never, 2–3 times/month, 1 time/week, 2–4 times/week, 5–6 times/week, and daily) were recorded for each food. Data were transformed to daily intake frequency. Portion sizes consumed from each food item were converted into grams, using standard Iranian household measures [20]. Daily food consumption was computed by multiplying the daily frequency of intake by portion size for each food item. Daily intakes of food (g/day) were then transferred to Nutritionist IV to calculate the total energy and nutrient intakes. To identify dietary patterns, the individual items were classified into 32 different categories based on similarity in nutrients, as shown in Table 1.

**Table 1:** Food grouping used in dietary pattern analysis

Food items	Food groups
Sausages, kalbas	Processed meats
Beef, lamb	Red meat
Beef liver, kale pache, maghz, zaban, sirabi	Organ meat
Canned tuna fish, other fish	Fish
Chicken with or without skin	Poultry
Egg	Egg
Skim milk, low fat milk, low fat yogurt, kashk	Low fat dairy products
High fat milk, whole chocolate and cocoa milk, high fat yogurt, creamy yogurt, cream cheese, other cheese, cream, ice cream	High fat dairy
Tea	Tea
Melon, honeydew melon, watermelon, pear, apricot, cherries, apple, peach, nectarine, Japanese plums, fig, grape, kiwifruit, grapefruit, orange, persimmon, tangerine, pomegranate, date, plums, sour cherries, strawberry, banana, lime, sweet lemon, pineapple	Fruit
Lemon juice, all types of juice	Fruit juices
Lettuce, cucumber, fresh basil, mixed vegetables, squash, eggplant, celery, green peas, green beans, carrot, onion, cabbage, spinach, bell pepper, mushroom, turnip	Vegetable
Lentils, kidney beans, chickpeas, broad beans, soy beans, mung beans, split peas	Legume
Iranian dark breads, barely	Whole grain
White bread, baguette bread, rice, white flour, spaghetti, noodle, biscuit, vermicelli	Refined grain
Garlic	Garlic
Tomatoes, red sauce	Tomato
Boiled potato	Potato
Hamburger, pizza, French fries	Fast food
Cracker, potato chips, salty snacks	Salty snack
Walnut, all types of nuts	Nut
Mayonnaise	Mayonnaise
Raisins, dried berries, dried limes	Dried fruit
Muffins, other cakes, sugar, white granulated sugar, honey, jam, gaz (Iranian sweet) Hard candy, chocolates, caramel flan, donuts	Sugar, sweet, dessert
Butter, margarine, hydrogenated fats, animal fats, non-hydrogenated oils	Solid fat
Canola oil, colza oil, sunflower oil, flaxseed oil, cotton oil, sesame oil	Vegetable oil
Green olives, olive oil	Olive
Soft drinks	Soft drink
Pickles	Pickles
Salt	Salt
Dough	Yogurt drink
Coffee	Coffee

### Assessment of other variables

Anthropometric measurements were obtained based on standard methods. Weight was measured by a digital scale, to an accuracy of 100 g, with participants wearing light indoor clothing and bare feet. Height was measured using a tape meter, with participants standing without shoes. Body mass index (BMI) was calculated as weight (in kilograms) divided by the square of the height (in meters).

Demographic information was gathered through a self-administered questionnaire. The socioeconomic status (SES) of study participants was assessed using a self-reported questionnaire. Data included: education level of the participant and the head of their family (below high-school diploma; diploma; diploma to bachelor’s degree; higher than bachelor's degree); employment status of the participant and the head of their family (yes/no); family size (≤ 4/ > 4); and housing status (owner/tenant) and type (apartment/villa). Demographic characteristics were obtained by a general self-reported questionnaire. These included: disease and drug history, medical history, and special diet and/or food prohibitions.

### Statistical analysis

For all statistical analyses, SPSS (version 21.0, SPSS Inc., Chicago, Illinois, USA) was used. Quantitative data were presented as mean and standard deviation, while qualitative variables were presented as frequency (numbers and percentages). To check the normality of the quantitative variables, the Kolmogorov–Smirnov test was used. KMO and Bartlett's Tests were applied to evaluate the adequacy of sample size and the applicability of the factor analysis method. Dietary patterns were derived using Principal Component Analysis (PCA) based on 168 food items. The individual items were classified into 32 predetermined different categories, based on similarity in nutrients. A scree diagram was used to determine the number of factors (dietary pattern) with an eigenvalue of > 0.2. Varimax rotation was applied on extracted factors (dietary patterns) in order to review correlations between variables and factors. The factor score for each dietary pattern was calculated by multiplying the number of nutrients by the pattern in the estimation of parameter; then each participant received a factor score for each specific pattern. All participants were categorized according to the median values of the dietary pattern scores.

To compare variables between cases and controls, independent samples *t*-test or chi-square test was applied, based on nature of the data. In addition, a one-way analysis of variance (one-way ANOVA) and chi-square test were used to compare means of quantitative and qualitative variables between the medians of each dietary pattern, respectively. Multivariable logistic regression was carried out to study the associations of dietary patterns with the odds of RA. First, in the crude model, the risk of RA was calculated between the medians of the identified dietary patterns. Then in the adjusted models, the following relationships were calculated: in Model I, adjustments were made for sex (male/female), BMI (kg/m^2^) and age (year). Model II additionally adjusted for disease history and lifestyle variables, including drug use, education, smoking (yes/no), physical activity (MET_min/wk), and socioeconomic status (low, moderate, high). Model III additionally adjusted for energy intake (kcal/day), and macronutrients. The significance level was considered to be 0.05 in this study.

## Results

The two major dietary patterns identified were: Healthy Dietary Pattern (including fish, egg, poultry, low-fat dairy products, fruit, etc.); and Western Dietary Pattern (including processed meats, red meat, organ meats, etc.). These patterns were revealed by factor analysis, as presented in Table 2.

**Table 2:** Factor-loading matrix for major dietary patterns

Food groups	Dietary patterns	
	Diet 1	Diet 2
Processed meats	–	0.355
Red meat	–	0.347
Organ meat	–	0.331
Fish	0.451	–
Poultry	0.241	–
Egg	0.532	–
Low fat dairy products	0.423	–
High fat dairy products	–	0.394
Tea	–	0.221
Fruit	0.703	–
Fruit juices	0.439	–
Vegetable	0.598	–
Legume	0.297	–
Whole grain	0.281	–
Refined grain	–	0.653
Garlic	–	0.641
Tomato	0.622	–
Potato	0.243	–
Fast food	–	0.867
Salty snack	–	0.409
Nut	–	0.433
Mayonnaise	–	0.326
Dried fruit	0.493	
Sugar	–	0.265
Solid fats	–	0.735
Vegetable oil	–	0.457
Olive	0.209	–
Soft drinks	–	0.255
Pickles	–	0.274
Salt	–	0.343
Yogurt drink	–	0.260
Coffee	–	–

The characteristics of participants in the case and control groups are presented in Table 3. There were no significant differences in terms of gender and energy intakes between cases and controls. However, age, smoking status, BMI, disease history, and MET in patients with RA were significantly higher (*P* < 0.001 for all). SES, as well as education, were significantly lower than those in the control group.

**Table 3:** Baseline characteristics of participants

Variables	Case	Control	*P *value
Age (year)	49.26 ± 12.6	40.88 ± 9.72	< 0.001
Sex, female (%)	81%	76.6%	0.37
BMI (kg/m^2^)	26.2 ± 4.35	24.82 ± 3.2	0.006
Socioeconomic status (SES)^b^	11.94 ± 3.47	13.57 ± 3.45	< 0.001
*Cigarette*			
No	83%	98%	< 0.001^a^
Yes	17%	2%	
*Disease history*			
No	41%	95.9%	< 0.001^a^
Yes	59%	4.09%	
*Drug use*			
No	58%	99%	< 0.001^a^
Yes	42%	1%	
*Education*			
Lower than diploma	50%	14.7%	< 0.001^a^
Diploma	33%	37%	
Bachelor	15%	31.5%	
Higher than bachelor	2%	16.8%	
Energy intake (kcal)	2243.02 ± 598.55	2168 ± 687.3	0.365
Carbohydrate (g)	258.29 ± 82	317.1 ± 116.63	0.015
Protein (g)	62.09 ± 18.49	72.59 ± 28.24	< 0.001
Fat (g)	98.57 ± 28	73 ± 32.32	< 0.001
Physical activity (MET-min/week)	4512.29 ± 6160	2372.74 ± 2055	< 0.001

Quantitative variables are shown as mean ± SD, and qualitative variables as frequency (percentage); *SD *standard deviation, *CI *confidence interval, *BMI *body mass index. *P *values are calculated by independent *t*-test

^a^For categorical data, *P *values are assessed by Chi-square test

^b^Socioeconomic status (SES) score was evaluated based on education level of both subjects and the family head, job of both subjects and the family head, family size, home status and home type by using self-reported questionnaire

The characteristics of the study participants across the dietary pattern median scores are presented in Table 4. There was a significant difference between case and control groups, with higher median healthy dietary pattern scores, correlating with age, weight, fat, and protein. Similarly, several residual variables, including age, weight, BMI and fat intake, also varied between case and control participants who scored above the median of the western dietary pattern.

**Table 4:** Characteristics and dietary intakes of study participants across median of dietary pattern scores

Variables	Healthy dietary pattern			Western dietary pattern		
	Case	Control	*P *value	Case	Control	*P *value
Age	46.28 ± 12.8	39.60 ± 9.2	< 0.001	49.2 ± 12.6	35.1 ± 7.5	< 0.001
Sex, female (%)	79.2%	71.6%	0.136	83.5%	77.4%	0.212
BMI	25.7 ± 4.44	24.5 ± 3.21	0.056	26.20 ± 4.35	24.2 ± 3.95	0.009
Weight	68.8 ± 12.21	63.7 ± 8.9	0.005	67.6 ± 11.4	62.2 ± 9.32	0.004
SES	12.8 ± 3.4	13.9 ± 3.8	0.134	13.3 ± 3.6	14.1 ± 3.3	0.255
*Disease history*						
No	76.5%	81.3%	0.281	74.7%	77.6%	0.316
Yes	23.5%	18.7%		25.3%	22.4%	
*Drug use*						
No	84.7%	93.2%	0.092	84.1%	88.1%	0.102
Yes	15.3%	6.8%		15.9%	11.9%	
*Education*						
< Diploma	30.3%	25.8%	0.116	28.7%	27.1%	0.521
Diploma	37.8%	35.6%		35.6%	38.3%	
Bachelor	23.5%	28.2%		24.8%	25.9%	
> Bachelor	8.4%	10.4%		10.9%	8.7%	
Physical activity (MET-min/week)	3631 ± 5145	2557 ± 2304	0.080	3512 ± 3480	3813 ± 2544	0.447
Energy (kcal)	2643 ± 509	2474 ± 707	0.137	2243 ± 598	2039 ± 712	0.069
Carbohydrate (g)	333 ± 76	359 ± 123	0.173	285.5 ± 82	280 ± 107	0.758
Protein (g)	73.1 ± 18.1	84.1 ± 29.9	0.017	64.5 ± 22.6	70.8 ± 28.4	0.694
Fat (g)	117 ± 24.5	84 ± 35.3	< 0.001	98.5 ± 28	79.4 ± 44.7	0.002
*Cigarette*						
No	91.2%	94.2%	0.8	94.8%	90.8%	0.83
Yes	8.8%	5.8%		5.2%	9.2%	

Multivariable adjusted OR for RA across the median values of the main dietary pattern scores are presented in Table 5. Lower adherence to the healthy dietary pattern increased the probability of RA 2.80 times in the crude model (OR = 2.80, 95% CI 1.74–4.67, *P* < 0.001). When the model was controlled for age, BMI and sex, the association remained significant (OR = 3.08, 95% CI 1.76–5.36, *P* < 0.001). Furthermore, after adding disease history, drug use, education, smoking, physical activity, and socioeconomic status in Model II (OR = 2.49, 95% CI 1.18–5.24, *P* = 0.016) the association remained significant. Also, after taking other confounders into account in Model III, the association still remained robust (OR = 2.85, 95% CI 1.12–7.45, *P* = 0.030). No statistically significant association was seen between western dietary pattern score and RA risk in the crude model (OR = 1.42, 95% CI 0.61–3.29, *P* = 0.40). However, after adjustment for age, sex and BMI in Model I, and after adding disease history, drug use, education, smoking, physical activity, and socioeconomic status in Model II, the association between western dietary pattern and RA altered, becoming a marginally significant association: Model I (OR = 1.56, 95% CI 0.88–2.75, *P* = 0.12); Model II (OR = 1.84, 95% CI 0.91–3.72, *P* = 0.08). In the final fully adjusted Model III, a positively significant relationship was found between western dietary pattern and RA (OR = 2.22, 95% CI 1.04–4.72, *P* = 0.03).

**Table 5:** Multivariable adjusted OR for RA across the median values of the main dietary pattern scores

Models	Healthy dietary pattern			Western dietary pattern		
	< Median	> Median	*P *value	< Median	> Median	*P *value
Crude	2.80 (1.74–4.67)	1	< 0.001	1	1.42 (0.61–3.29)	0.40
Model I	3.08 (1.76–5.36)	1	< 0.001	1	1.56 (0.88–2.75)	0.12
Model II	2.49 (1.18–5.24)	1	0.016	1	1.84 (0.91–3.72)	0.08
Model III	2.85 (1.12–7.45)	1	0.030	1	2.22 (1.04–4.72)	0.03

Model 1: Adjusted for age, sex and BMI

Model 2: Adjusted for model 1 plus disease history, drug use, education, smoking, physical activity, and socioeconomic status

Model 3: Adjusted for model 1 plus energy and macronutrient intakes

## Discussion

In this case–control study, two major dietary patterns were identified: healthy dietary pattern and western dietary pattern. According to the results, those with lower adherence to healthy dietary pattern had a higher risk of RA. However, the western dietary pattern was positively associated with the disease. Limited data is available regarding broad dietary patterns and their relationships to RA in Asiatic and especially Iranian populations, so the present findings can help develop a better understanding of the relationship between diet and this chronic disease.

RA has an adverse influence on patients’ abilities to perform normal daily activities, and can reduce both employment opportunities and quality of life. RA imposes substantial economic burdens on individuals and societies [21, 22]. In the present study, a significant protective association was found between healthy dietary pattern and the likelihood of RA. A number of empirical studies have shown that a healthier diet including fruits, vegetables, whole grains, nuts, long chain (n-3) fat, and polyunsaturated fat was related to a reduced risk of RA [23, 24]. The present results are consistent with another study which reported a reduced risk of RA (particularly seropositive RA) following a healthier dietary pattern in women aged ≤ 55 [23]. In another population-based case–control study, Mediterranean diet was inversely associated with RA risk among men, and only in seropositive RA [15]. There is a strong similarity between the content of the healthy dietary pattern in the current study, and a Mediterranean dietary pattern. In the present study, fish and fruit were a part of the healthy dietary pattern, and had previously been shown to correlate with reduced RA risk in previous studies [13, 24, 25]. The probable protective role of fish against RA could be due to its high content of n-3 polyunsaturated fatty acids. Long-chain omega-3 fatty acids are among the most potent edible inflammation fighters, and could protect against RA through their anti-inflammatory properties. Also, fish oil consumption has the potential to reduce the arachidonic acid content of cell membranes, thus resulting in lower production of proinflammatory chemicals from arachidonic acid [26, 27]. Findings from studies investigating the impact of fish intake on RA risk remain inconsistent, however [12, 25, 28]. Although a specific reason has not yet been identified, these differences may be due to an imbalance between the protective effect of long-chain n-3 PUFAs and contaminants that are positively related to RA risk, such as polychlorinated biphenyls (PCBs) [29].

Also fruits, dried fruits, and vegetables are major components in a healthy diet. Several studies have shown the protective effects of fruit and vegetable consumption on RA [12, 25]. Fruits and vegetables are good sources of various micronutrients, such as minerals, antioxidants, and fiber, with some of them having well-known anti-inflammatory effects [30]. Free oxygen radicals and proinflammatory cytokines are factors involved in tissue damage in RA. Antioxidants are parts of a healthy diet, and can protect against reactive oxygen species [13].

The intake of low-fat milk and dairy products has also been shown to decrease the risk of RA. A possible mechanism of this effect may be attributed to the micronutrients in milk, such as riboflavin, calcium, and vitamin B12, and also to the immunomodulating effects of vitamin D [31].

Previous studies have shown that foods and diets perhaps play a role in the cause of RA through altering systemic inflammation and immune-inflammatory responses [32]. It has been documented that western dietary patterns, containing higher intake of high-protein, red meat, processed meat, and refined grains, as well as frequent consumption of processed and fast foods, could promote the risk of diseases related to enhanced inflammatory factors such as diabetes, metabolic syndrome and autoimmune diseases [33, 34].

A number of studies have suggested that high levels of red meat consumption are a risk factor for the development of inflammatory polyarthritis and RA [14, 35]. Meat products (especially red meat) and full-fat dairy products are major sources of saturated fats in unhealthy dietary patterns, which may promote adipose-induced inflammation [30]. Additionally, red meat is a source of arachidonic acid, a pro-inflammatory eicosanoid [14].

The human body needs a healthy balance of the n-6/n-3 ratio. Excess consumption of omega-6 s can influence the function of adipose tissue and promote the production of pro-inflammatory chemicals [30, 36–38]. One study has reported that the regular consumption of sugar-sweetened soda is associated with increased risk of seropositive RA in women [10]. The results obtained from several studies have shown a higher prevalence of metabolic syndrome and other metabolic disorders, such as obesity, dyslipidemia, or impaired glucose metabolism, in RA patients. There seems to be sufficient evidence to support the hypothesis that there is a relationship between insulin resistance and inflammation, and RA, which is similar to other chronic diseases [39]. On the other hand, the positive relationship between the sweet/desserts group and RA risk can be ascribed to its high processed sugar and trans-fat content. These, usually found in cookies, donuts, and cakes, stimulate the release of cytokines and trigger systemic inflammation. Due to high calorie content without any fiber or nutrients, they can lead to weight gain [30].

In contrast, some studies have reported no significant association between diet and RA [40]. These contradictory results might arise from differences in study design, study populations, and statistical and data collection methods (especially relating to dietary intakes and adjusted potential confounders).

This study has many strengths. Newly diagnosed patients were selected to reduce the likelihood of post-diagnostic dietary changes. The effects of several potential confounders were controlled for in the statistical analyses. Using principal component analysis can lead to increased power in detecting diet-disorder associations by creating continuous variables. However, the present study also possesses a number of limitations, including a relatively small sample size, and the evaluation of dietary patterns using a self-reported 1-year FFQ (which may increase the possibility of errors in measuring dietary intake). It is possible that patients had changed their diets after RA diagnosis, thus the present findings may not reflect the accurate relation between diet and RA risk. To limit this potential for inaccuracy, cases were recruited from newly diagnosed patients in order to collect food intake data before extensive modifications to habitual dietary patterns. In addition, some of the variables measured were different between cases and controls across the dietary patterns, which might have influenced the findings. However, this problem was tackled by adjusting these variables to mitigate them. Also, some potential confounders were not assessed, such as stress level and genetic factors, which might have influenced the results.

## Conclusion

The present study suggests that healthy dietary pattern is associated with a reduced likelihood of RA, and the western dietary pattern appears to be associated with increased odds of RA among Iranians. This presents new insights with regard to dietary control and managing RA risk. However, more studies are required in this area to confirm the present findings.

## Data Availability

The datasets generated and analyzed during the current study are not publicly available, due to institutional policy, but may be requested directly from the corresponding author.

## Abbreviations

RA: Rheumatoid arthritis
FFQ: Food frequency questionnaire
HDP: Healthy dietary pattern
WDP: Western dietary pattern
